# Clinical and molecular correlates from a predominantly adult cohort of patients with short telomere lengths

**DOI:** 10.1038/s41408-021-00564-7

**Published:** 2021-10-22

**Authors:** Abhishek A. Mangaonkar, Alejandro Ferrer, Filippo Pinto E. Vairo, Caleb W. Hammel, Carri Prochnow, Naseema Gangat, William J. Hogan, Mark R. Litzow, Steve G. Peters, J. P. Scott, James P. Utz, Misbah Baqir, Eva M. Carmona-Porquera, Sanjay Kalra, Hiroshi Sekiguchi, Shakila P. Khan, Douglas A. Simonetto, Eric W. Klee, Patrick S. Kamath, Anja C. Roden, Avni Y. Joshi, Cassie C. Kennedy, Mark E. Wylam, Mrinal M. Patnaik

**Affiliations:** 1 Division of Hematology, Department of Medicine, Rochester, MN USA; 2 Center for Individualized Medicine, Department of Quantitative Health Science, Rochester, MN USA; 3 Department of Clinical Genomics, Rochester, MN USA; 4 Division of Pulmonary and Critical Care Medicine, Department of Medicine, Rochester, MN USA; 5 Division of Pediatric Hematology/Oncology, Department of Pediatrics, Rochester, MN USA; 6 Division of Gastroenterology, Department of Medicine, Rochester, MN USA; 7 Department of Laboratory Medicine and Pathology, Rochester, MN USA; 8 Division of Pediatric Allergy and Immunology, Department of Pediatrics, Mayo Clinic, Rochester, MN USA

**Keywords:** Cancer stem cells, Haematopoietic stem cells

**Dear Editor**,

Telomere biology disorders (TBDs) are accelerated aging syndromes affecting hematopoietic, pulmonary, hepatobiliary, and/or immunological systems among others [1, 2]. Adult-onset TBDs are commonly associated with pathogenic variants in *TERT* and *TERC*, and manifest with varying degree of organ involvement such as idiopathic interstitial pneumonia (IIP), cryptogenic cirrhosis, unexplained bone marrow failure (BMF) and/or immunodeficiency, and an inherent risk for cancer development such as myeloid leukemias and squamous cell carcinomas among others [3–9]. Though fluorescence in-situ hybridization (FlowFISH)-determined age-adjusted lymphocyte TL > 50 %tile have a 100% negative predicted value for variant detection, the TL threshold below which genetic screening should be pursued (especially in older patients) is still not known [10, 11]. The optimal TL threshold for variant detection depends on age, with individuals at ages <20 years showing strong correlations with lower TL, while the TL in older variant carriers often overlaps with the lower decile of normal controls [10]. Genetic screening is suggested for TL ≤ 10th centile in lymphocytes or granulocytes, especially in the presence of suggestive personal or family history of a TBD [1]. However, the utility of genetic testing for adult patients with TL > 10th centile and integration of clinical phenotype is currently unclear. Additionally, natural outcomes and diagnostic algorithms are less clear in adult (age ≥ 18 years) patients with short telomeres (defined as TL ≤ 10th centile in lymphocytes).

We retrospectively abstracted data from patients who underwent FlowFISH testing at our institution from years 2015 to 2020. All patients who underwent FlowFISH testing had a suspected clinical phenotype based on at least one or more of the salient clinical features mentioned below. FlowFISH assessments were done at reference laboratories in Vancouver (RepeatDx; Canada) and Johns Hopkins University (JHU, USA). In order to objectively quantify clinical risk, a clinical likelihood score (CLS) was developed based on the number of presenting clinical features suggestive of TBD (prior to FlowFISH testing), or the presence of a significant family history of the same in one or more 1st or 2nd degree relatives, with categories including low (none or 1), intermediate (2), or high risk (>2), respectively (Supplementary Methods). Salient clinical features were pre-determined as, personal history of premature hair graying (onset at age <30 years), idiopathic interstitial pneumonia (IIP), or IIP/emphysema overlap, cryptogenic cirrhosis or nodular regenerative hyperplasia (NRH), persistent unexplained cytopenias [defined as low blood counts in one or more cell lineages (red or white blood cell or platelets), persistent for 6 months or longer], and/or immunodeficiency.

Genetic testing was performed using either an in-house research-based whole exome sequencing (WES) or commercial bone marrow failure-specific targeted next generation sequencing (NGS) panel or exome-based customized panels (ES-Slices) (Supplementary table 1). Whole-exome sequencing (WES) was performed at the Clinical Genomics Laboratory (Mayo Clinic) using a previously published protocol [12, 13]. Genomic data was processed through an in-house bioinformatics pipeline and analyzed by the Translational Omics Program at the Center for Individualized Medicine (Mayo Clinic) using Emedgene analysis software (Emedgene Technologies).

Two hundred and fifty-two patients at our institution underwent TL assessment at RepeatDx (*n* = 71) and JHU (*n* = 181) laboratories. Median age was 57 (range: 4–83) years; 144 (57%) being males; 236 (94%) adults. Significant family history was present in 66 (26%) patients, while premature graying of hair was present in 24 (10%) patients. Organ-specific clinical features included cytopenias (*n* = 117, 46%), IIP (*n* = 135, 54%), gastrointestinal disease [n = 38 (15%), cryptogenic cirrhosis-30, NRH-4, enteropathy-2, steatosis-2], and immunodeficiency (*n* = 37, 15%). Patterns of IIP included 41 (30%) usual interstitial pneumonia (UIP), 15 (11%) non-specific interstitial pneumonia (NSIP), 9 (7%) chronic hypersensitivity pneumonitis (CHP), 2 (1.5%) lymphocytic interstitial pneumonitis and 1 desquamative interstitial pneumonia. History of smoking was present in 80 (32%) patients, with a median of 24 (0.5–210) pack years of smoking and correlated with IIP (*p* = 0.01). Emphysematous changes were seen in 15 (11%) patients; 67% in smokers [median, 29 (2.5–70) pack years]. Pulmonary function test information was available in 148 [59%, 108 (80%) in patients with pulmonary disease]. The %predicted values of FEV1 [median, 63.5 versus 92, *P* < 0.0001] and FVC [median 61.1 versus 98.5, *P* < 0.0001] were lower, while the FEV1/FVC ratio (median 106.7 versus 93.7, *P* < 0.0001) was higher in patients with versus without IIP [defined by high-resolution computed tomography (HRCT) imaging findings [14]]. The clinical diagnoses for patients before TL testing included 107 (42%) IIP, 22 (9%) aplastic anemia, 13 (5%) each with unexplained pancytopenia, immunodeficiency, and IIP/cirrhosis, 12 (5%) cirrhosis, 11 (4%) common variable immunodeficiency (CVID), 8 (3%) each with BMF and lymphopenia, 5 (2%) each with unexplained anemia, bicytopenia, and myelodysplastic syndrome, 4 (2%) each with IIP/CVID and neutropenia, 3 (1%) with thrombocytopenia, 2 (<1%) each with hypersensitivity pneumonitis (HP) and NRH, 1 (<1%) each with CVID/NRH, acute myeloid leukemia, Diamond-Blackfan Anemia, IIP/BMF, IIP/cirrhosis/BMF, IIP/NRH/BMF, IIP/pancytopenia, and leukopenia. Seven (3%) patients were tested only due to a significant family history without an obvious clinical phenotype.

CLS stratification included low (*n* = 139, 55%), intermediate (*n* = 83, 33%), and high (*n* = 30, 12%) groups, with higher CLS significantly correlating with lower delta (age-adjusted) TL for lymphocytes (*P* = 0.01) but not granulocytes (*P* = 0.1). Genetic testing was performed in 82 (33%) patients (targeted NGS panel-31, ES-Slice-39,WES-12) which was positive for 9 (11%) pathogenic or likely pathogenic (8 *TERT* and 1 *RTEL1*) variants and 16 (19.5%) variants of uncertain significance (Supplementary table 2). Since this was a retrospective study, the genetic analyses are limited by the fact that certain patients were more likely to be tested than others based on clinical features. Among patients with short telomeres (TL in lymphocytes ≤10%, *n* = 158, 146 adults), only 17% (9/54 tested patients) were positive for pathogenic variants. This suggests potential alternative, epigenetic or non-genetic mechanisms of telomere shortening/telomere length regulation such as aging-associated methylation and/or chromatin modifications, and environmental factors as suggested in other studies [15, 16]. ‘High risk’ per CLS score (as defined above) was unable to predict likelihood of finding a telomere-associated variant (*P* = 0.6), highlighting the shortcomings in the predictive value of phenotypic changes/family histories in adult patients with suspected TBDs.

Based on age-appropriate centile categorization of lymphocyte and granulocyte TL (information for both available in 233 patients), patients were stratified into four groups (Table 1). Group A (*n* = 14) included TL < 1st centile in both lymphocytes and granulocytes. Among the 9 (64%) patients who underwent genetic testing (4 targeted NGS panels, 4 ES-Slice, and 1 WES) in this group, 4 (44%) patients were found to have pathogenic variant (all *TERT*) and 2 (22%) patients were detected to have a VUS (both *RTEL1*). Group B included patients with TL 1–10th centile in both lymphocytes and granulocytes, or <1st centile in either lymphocytes or granulocytes (*n* = 84). Twenty-nine (35%) underwent genetic testing (11 targeted NGS panels, 16 ES-Slice, and 2 WES) with 4 pathogenic variants (3 *TERT*, 1 *RTEL1*), and 5 VUS (2 *RTEL1*, 1 *TERT*, 1 *NHP2*, and 1 *PARN*). Group C included patients with TL 1–10th centile in lymphocytes only and >10th centile in granulocytes (*n* = 53). Thirteen (25%) patients in this group underwent genetic testing (2 NGS-panel, 7 ES-Slice, 4 WES) with 1 pathogenic variant (*TERT*) and 3 VUS (2 in *TINF2*, 1 in *RTEL1)*. Group D included patients with TL > 10th centile in lymphocytes (*n* = 82). Genetic testing was done in 22 (27%) patients (9 NGS-Panel, 8 ES-Slices, and 5 WES) of whom only 5 (23%) had VUS in *2 RTEL1,1 TINF2, 1 WRAP53*, and 1 *CTC1*, without any *bonafide* pathogenic variants (Fig. 1A and Table 1). In patients with pulmonary disease, %predicted values of FEV1 (*R*^2^ = 0.08, *P* = 0.3), FVC (*R*^2^ = 0.1, *P* = 0.2), and FEV1/FVC (*R*^2^ = 0.1, *P* = 0.3) did not correlate with the likelihood of finding a telomere-associated pathogenic variant. Interestingly, one 47-year-old patient had FlowFISH testing (at the same laboratory) at three different time points in a span of 6 months (post-transplant), with results demonstrating variability in telomere length measurement (Supplementary table 3) and categorization, which could partly be due to the interassay coefficient of variation for FlowFISH (2.5% for lymphocytes and 2.1% for granulocytes) [10]. For practical clinical decision-making, data for additional categories such as patients with TL < 1st centile in lymphocytes and >1st centile in granulocytes, and 1–10th centile in lymphocytes, ≥1st centile in granulocytes is provided in Supplementary table 4.

**Table 1:** Table showing distribution of clinical features, genetic testing, and outcomes for the considered FlowFISH categories in the diagnostic assessment of patients (range or % only provided if *n* > 1).

Characteristic (median; % or range)	Total (*n* = 233)	FlowFISH TL centile categories (categorization possible in 233 patients)				*P* value
		Group A (<1st centile TL in both lymphocytes and granulocytes) (*n* = 14)	Group B (1-10th centile in both lymphocytes and granulocytes, or <1st centile in either lymphocytes or granulocytes) (*n* = 84)	Group C ( < = 10th centile in lymphocytes only) (*n* = 53)	Group D (>10th centile in lymphocytes) (*n* = 82)	
Age (in years)	58 (4–83)	50 (6–66)	60 (4–77)	59 (17–70)	49.5 (10–83)	**0.04**
No. of males (%)	144 (57)	40 (49)	56 (67)	31 (58)	40 (49)	0.1
Family history	63 (25)	4 (29)	28 (33)	13 (25)	18 (22)	0.4
Premature graying of hair (onset at age ≤30 years)	23 (10)	3 (21)	11 (13)	5 (9)	4 (5)	0.2
IIP	135 (54)	9 (64)	60 (71)	28 (53)	35 (43)	**0.002**
Cytopenias	103 (44)	7 (50)	39 (46)	18 (34)	39 (48)	0.4
Cirrhosis	28 (12)	1 (7)	16 (19)	2 (4)	9 (11)	**0.04**
NRH	4 (2)	1 (7)	1 (1)	-	2 (2)	0.3
Immunodeficiency	37 (16)	2 (14)	6 (7)	14 (26)	15 (18)	**0.02**
*Clinical likelihood of STS*^a^						
Low (1)	123 (53)	7 (50)	33 (39)	29 (55)	54 (66)	**0.008**
Intermediate (2)	81 (35)	4 (29)	33 (39)	20 (38)	24 (29)	0.5
High (>2)	29 (12)	3 (21)	18 (21)	4 (8)	4 (5)	**0.003**
Delta TL in lymphocytes (kb)	−1.12 (−5.11 to 2.8)	−2.73 (−5.11 to 2.62)	−1.62 (−3.28–0.7)	−1.4 (−2.5–1)	−0.07 (−1.13–2.8)	**<0.0001**
Delta TL in granulocytes (kb)	−1.27 (−10.6 to 6.35)	−2.8 (−4.1 to −1.9)	−1.84 (−10.6–2.29)	−1.1 (−2.5–5.72)	−0.4 (−3.4–6.4)	**<0.0001**
No. of patients with genetic testing^b^	73 (31)	9 (64)	29 (35)	13 (25)	22 (27)	**0.03**
*No. of patients with telomere-related variants*						
Pathogenic/likely pathogenic	9 (12)	4 (44)	4 (14)	1 (7)	–	**0.008**
VUS	16 (22)	2 (22)	5 (17)	3 (20)	5 (23)	0.9

This table includes data on 233 patients out of the total cohort of 252 patients. In 19 patients, data on both lymphocytes and granulocytes was not available for categorization into the TL categories. Significant clinical features for TBD considered were personal history of premature graying of hair (onset at age ≤ 30 years), IPF, unexplained cytopenias, cirrhosis or NRH, and unexplained immunodeficiency, or significant family history of the above (in one or more 1st or 2nd degree relatives).

*FlowFISH* flow cytometry fluorescence in-situ hybridization, *TL* telomere length, *IIP* idiopathic interstitial pneumonia, *NRH* nodular regenerative hyperplasia, *TBD* telomere biology disorders, *VUS* variant of uncertain significance.

^a^Based on the number of the significant clinical features, clinical likelihood score was defined as low (1), intermediate (2), and high (>2).

^b^Included patients were tested with panels designed to test bone marrow failure-related genes. In other words, patients who underwent genetic testing with hematologic malignancy-based next-generation sequencing panels were excluded.

*Bold values indicate statistical significance.*

**Fig. 1:**
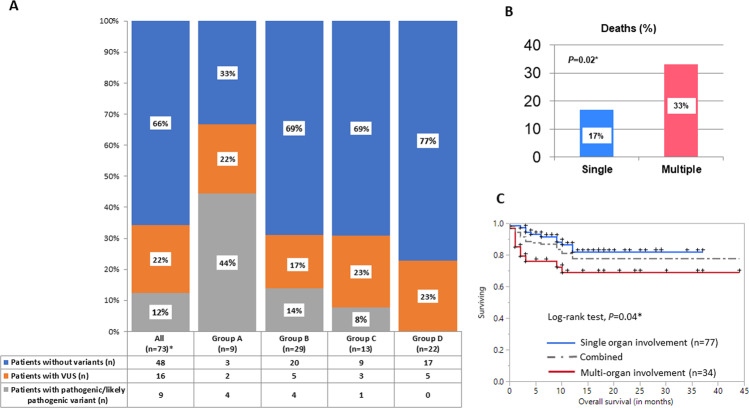
Figure showing genetic characteristics and clinical outcomes of patients with clinically-relevant short telomeres. **A** shows genetic testing information and the frequencies of pathogenic variants and variants of uncertain significance in different FlowFISH centile categories. **B** shows a higher frequency of deaths in patients with multiple organ involvement compared to single organ involvement (33% versus 17%, *P* = 0.02*) in patients with clinically-relevant short telomeres (telomere length ≤10^th^ centile in lymphocytes). **C** shows a higher Kaplan–Meier estimate of overall survival (OS) in untreated (non-transplanted) patients with short telomeres (telomere length ≤10th centile in lymphocytes) with single organ (*n* = 77) versus patients with multiple organ (*n* = 34) involvement (median not reached in either category, *P = 0.04).

We then assessed outcomes for patients with TL ≤ 10th centile in lymphocytes (*n* = 151). At a median follow-up of 15 (95% CI 13–17) months, 124 (78%) patients were alive with 34 (22%) deaths; higher frequency in non-transplanted (untreated) patients with multiple organ involvement compared with single organ involvement (33 versus 17%, *P* = 0.02, Fig. 1B). The median Kaplan–Meier estimate of overall survival computed from the time of FlowFISH testing was not reached. When patients were categorized by organ involvement, those with multiple organ involvement had a worse Kaplan–Meier estimate of overall survival (OS, median not reached, *P* = 0.04), with similar findings for untreated (*n* = 115) patients (median not reached, *P* = 0.03, Fig. 1C) and no differences among patients who underwent organ-specific transplantation (supplementary fig. 1).

Our study demonstrates the importance of using a Flow-FISH assay based predictive algorithm to screen adult patients with suspected STS for telomere-related genetic alternations. We also demonstrate the limited role for genetic testing in adult patients with lymphocyte TL > 10th centile, regardless of the clinical likelihood. Adult patients with shortened telomere lengths have a <20% positivity rate for a TBD-associated pathogenic variant suggesting alternative, epigenetic and/or non-genetic mechanisms of telomere length regulation. Potential caveats of using FlowFISH testing include interassay variability, measuring only mean TL, lack of ability in measuring tissue-specific TL, and possibility of missing silent genetic carriers who may have TL at the lower end of the normal range. Patients with multiple organ involvement clearly have worse outcomes, with multiorgan transplant strategies available at select centers.

## Supplementary information

Supplementary methods

Supplementary table 1

Supplementary table 2

Supplementary table 3

Supplementary table 4

Supplementary figure 1
