# Supplementary material for: Clinical and molecular correlates from a predominantly adult cohort of patients with short telomere lengths
Source: Blood Cancer J. 2021 Oct 22;11(10):170. doi: 10.1038/s41408-021-00564-7 (PMC8536738; doi:10.1038/s41408-021-00564-7)
Supplement: Supplementary file 1 — Supplementary methods [file 41408_2021_564_MOESM1_ESM.docx]

**Supplementary methods**

### Whole Exome Sequencing

Whole exome sequencing (WES) was performed at the Clinical Genomics Laboratory (Mayo Clinic) using a standard procedure summarized as follows: paired-end libraries were prepared using 1.0 µg of genomic DNA using the Agilent Bravo liquid handler (Agilent) as indicated by the manufacturer. Whole exon capture was carried out using 750 ng of the prepped library following the protocol for Agilent’s SureSelect Human All Exon v5 + UTRs 75 MB kit. The purified capture products were amplified using the SureSelect Post-Capture Indexing forward and Index PCR reverse primers (Agilent) for 12 cycles. The concentration and size distribution of the completed captured libraries was determined on Qubit (Invitrogen) and Agilent Bioanalyzer DNA 1000 chip.

Libraries were sequenced at an average coverage of ~80X following Illumina’s standard protocol in an Illumina cBot and HiSeq 3000/4000 PE Cluster Kit. The flow cells were sequenced as 150 X 2 paired end reads on an Illumina HiSeq 4000 using HiSeq 3000/4000 sequencing kit and HCS v3.3.52 collection software. Base-calling is performed using Illumina’s RTA version 2.7.3.

Genomic data was processed through an in-house bioinformatics pipeline and analyzed by the Translational Omics Program at the Center for Individualized Medicine (Mayo Clinic) using Emedgene analysis software (Emedgene Technologies).

**Clinical likelihood score (CLS)***

Clinical likelihood score (CLS) was defined based on the number of clinical features. Clinical likelihood score (CLS) was assigned as low (none or 1), intermediate (2) or high risk (>2), based on the number of clinical features present prior to FlowFISH testing. Salient clinical features were pre-determined as, personal history of premature hair greying (onset at age < 30 years), idiopathic interstitial pneumonia (IIP) or IIP/emphysema overlap, cryptogenic cirrhosis or nodular regenerative hyperplasia (NRH), cytopenias and/or immunodeficiency, and family history of the above (in one or more 1^st^ or 2^nd^ degree relatives).

*This score was designed to objectively quantify the clinical suspicion of a telomere phenotype in order to enable comparison with a telomere length directed approach of diagnosis short telomere syndrome (STS). It is not validated for diagnosis of a short telomere syndrome.
