# Supplementary material for: Clinical and molecular correlates from a predominantly adult cohort of patients with short telomere lengths
Source: Blood Cancer J. 2021 Oct 22;11(10):170. doi: 10.1038/s41408-021-00564-7 (PMC8536738; doi:10.1038/s41408-021-00564-7)
Supplement: Supplementary file 2 — Supplementary table 1 [file 41408_2021_564_MOESM2_ESM.docx]

**Supplementary table 1:** Details of next generation sequencing testing including telomere-related genes used to test the cohort.

| **Gene panel** | **Institution** | **List of genes assessed** | **Coding region coverage** | **Read depth** | **Number of individuals tested** |
| --- | --- | --- | --- | --- | --- |
| Custom XomeDxSlice | GeneDX (designed by Mayo Clinic) | *ABCB7; ABCG5; ABCG8; ACD; ACTN1; ADA; ADAMTS13; AK2; ALAS2; ANKRD26; AP3B1; ATM; ATR; BLM; BLOC1S6; BRCA1; BRCA2; BRIP1; C15orf41; C3; CARD11; CASP10; CASP8; CBL; CD27; CD3D; CD3E; CD40LG; CD46; CDAN1; CEBPA; CFB; CFH; CFHR1; CFHR3; CFHR4; CFHR5; CFI; CHEK2; CLPB; CSF3R; CTC1; CTLA4; CXCR2; CXCR4; CYCS; DCLRE1B; DCLRE1C; DDX41; DGKE; DKC1; DNAJC21; DNMT3B; DOCK8; ELANE; EPO; ERCC4; ERCC6L2; ETV6; FADD; FANCA; FANCB; FANCC; FANCD2; FANCE; FANCF; FANCG; FANCI; FANCL; FANCM; FAS; FASLG; FLI1; FLNA; FOXN1; FOXP3; FYB1; G6PC3; GAR1; GATA1; GATA2; GFI1; GFI1B; GLRX5; GP1BA; GP1BB; GP9; HAX1; HOXA11; IKZF1; IL2RG; IL7R; ITGA2B; ITGB3; ITK; JAGN1; JAK2; JAK3; KDM1A; KLF1; KRAS; LAMTOR2; LIG4; LRBA; LYST; MAD2L2; MAGT1; MECOM; MLH1; MPL; MSH2; MSH6; MYH9; NAF1; NBEAL2; NBN; NF1; NHEJ1; NHP2; NOP10; NPAT; NPM1; NRAS; ORAI1; PALB2; PARN; PAX5; PGM3; PIK3CD; PMS2; PNP; POT1; PRF1; PRKACG; PTPN11; PTPRC; PUS1; RAB27A; RAC2; RAD50; RAD51; RAD51C; RAG1; RAG2; RBM8A; RECQL4; RFWD3; RPL11; RPL15; RPL26; RPL27; RPL35; RPL35A; RPL5; RPS10; RPS15A; RPS17; RPS19; RPS24; RPS26; RPS27; RPS28; RPS29; RPS7; RTEL1; RUNX1; SAMD9; SAMD9L; SBDS; SEC23B; SH2D1A; SIRT1; SIRT4; SIRT5; SLC19A2; SLC25A38; SLC37A4; SLC7A7; SLFN14; SLX4; SRC; SRP54; SRP72; STAT3; STAT5B; STIM1; STK4; STN1; STX11; STXBP2; TAZ; TBX1; TCIRG1; TCN2; TERC; TERF1; TERF2IP; TERT; THBD; THPO; TINF2; TNFRSF13B; TP53; TRNT1; TSR2; TUBB1; UBE2T; UNC13D; USB1; VHL; VPS13B; VPS45; VWF; WAS; WIPF1; WRAP53; XIAP; XRCC2; YARS2; ZAP70; ZCCHC8; EFL1; PAPD5* | 100% (Except IKZF1: 10.3%) | 100-120X | 39 |
| Bone Marrow Failure Gene Sequencing Panel | Cincinnati Children’s Hospital Medical Center | *AP3B1; BRCA2; BRIP1; CSF3R; CXCR4; DKC1; ELANE; ERCC4; FANCA; FANCB; FANCC; FANCD2; FANCE; FANCF; FANCG; FANCI; FANCL; FANCM; G6PC3; GATA1; GATA2; GFI1; HAX1; LAMTOR2; LYST; MPL; NHP2; NOP10; PALB2; RAB27A; RAC2; RAD51C; RBM8A; RMRP; RPL5; RPL11; RPL15; RPL26; RPL35A; RPS7; RPS10; RPS17; RPS19; RPS24; RPS26; RTEL1; SBDS; SLC37A4; SLX4; SRP72; TAZ; TERC; TERT; TINF2; USB1; VPS13B; VPS45; WAS; WIPF1; WRAP53* | 100% | >20X | 11 |
| Dyskeratosis Congenita Gene Sequencing Panel | Cincinnati Children’s Hospital Medical Center | *DKC1; NHP2; NOP10; RTEL1; TERC; TERT; TINF2; WRAP53* | 100% | >20x | 6 |
| Telomere Defect Gene Panel | Mayo Clinic Laboratories | *CTC1; DKC1; NHP2; NOP10; RTEL1; TERC; TERT; TINF2; USB1; WRAP53* | 100% | >20x | 7 |
| Telomere Shortening Disorders Spectrum NGS Panel | The Johns Hopkins Hospital | *CTC1;DKC1; NHP2; NOP10; TERC; TERT; TINF2; WRAP53* | 100% | >50X | 2 |
