# Supplementary material for: Clinical and molecular correlates from a predominantly adult cohort of patients with short telomere lengths
Source: Blood Cancer J. 2021 Oct 22;11(10):170. doi: 10.1038/s41408-021-00564-7 (PMC8536738; doi:10.1038/s41408-021-00564-7)
Supplement: Supplementary file 3 — Supplementary table 2 [file 41408_2021_564_MOESM3_ESM.docx]

**Supplementary Table 2. Detailed information of genetic variants identified in this study.**

| Gene | Genomic alteration (GRCh38) | cDNA | Predicted Protein Change | CADD score | gnomAD frequency (total population) | ACMG Classification by clinical laboratory | Genetic testing |
| --- | --- | --- | --- | --- | --- | --- | --- |
| CTC1 | chr17:8238597T>C | c.230A>G | p.(Gln77Arg) | 25 | Not present | VUS | Cinncinnati NGS |
| NAF1 | chr4:163140337C>T | c.764G>A | p.(Arg255Gln) | 26.4 | 0.00041% | VUS | BMF XomeDxSlice GeneDx |
| NHP2 | chr5:178149715_178149716del | c.459_460delAT | p.(*154Argext*29) | N/A | Not present | VUS | BMF XomeDxSlice GeneDx |
| PARN | chr16:14627162A>T | c.271T>A | p.(Tyr91Asn) | 27.9 | 0.0025% | VUS | Invitae PID panel |
| *PARN | chr16:14628181C>G | c.168G>C | p.(Lys56Asn) | 23.1 | 0.0025% | VUS | BMF XomeDxSlice GeneDx |
| RTEL1 | chr20:63690334G>A | c.2378G>A | p.(Arg793His) | 15.1 | 0.0086% | VUS | Telomere gene defects panel |
| RTEL1 | chr20:63690164_63690172del | c.2291_2299delATGTCATCC | p.(His764_Ile766del) | N/A | Not present | Pathogenic | BMF XomeDxSlice GeneDx |
| RTEL1 | chr20:63659503A>G | c.101A>G | p.(Gln34Arg) | 33 | Not present | VUS | Cinncinnati NGS |
| RTEL1 | chr20:63690194G>A | c.2321G>A | p.(Arg774His) | 32 | 0.00040% | VUS | Telomere gene defects panel |
| RTEL1 | chr20:63690938C>T | c.2619C>T | p.(Gly873=) | 5.815 | 0.0026% | VUS | Invitae PID panel |
| *RTEL1 | chr20:63695197C>T | c.3547C>T | p.(Leu1183Phe) | 12.24 | Not present | VUS | BMF XomeDxSlice GeneDx |
| RTEL1 | chr20:63672619G>A | c.835G>A | p.(Val279Met) | 25.4 | 0.0039% | VUS | BMF XomeDxSlice GeneDx |
| TERC | chr3:169764670C>T | n.61C>T | N/A | 9.528 | N/A | VUS | Telomere gene defects panel |
| TERT | chr5:1260497G>A | c.2947C>T | p.(His983Tyr) | 25.5 | Not present | Likely Pathogenic | Research panel |
| TERT | chr5:1293547G>C | c.1339C>G | p.(Arg447Gly) | 6.554 | Not present | VUS | WES |
| TERT | chr5:1280223C>T | c.1885 G>A | p.(Gly629Arg) | 23.7 | Not present | Pathogenic | BMF XomeDxSlice GeneDx |
| **TERT | chr5:1264479G>A | c.2768C>T | p.(Pro923Leu) | 24.6 | 0.00040% | Likely Pathogenic | Cinncinnati NGS |
| **TERT | chr5:1279391C>T | c.2030G>A | p.(Gly677Asp) | 23.8 | Not present | Likely Pathogenic | BMF XomeDxSlice GeneDx |
| TERT | chr5:1282433T>G | c.1765A>C | p.(Ile589Leu) | 5.061 | Not present | Likely Pathogenic | BMF XomeDxSlice GeneDx |
| TERT | chr5:1253765G>A | c.3362C>T | p.(Pro1121Leu) | 24.2 | Not present | Likely Pathogenic | BMF XomeDxSlice GeneDx |
| TINF2 | chr14:24240300C>T | c.1092G>A | p.(Leu364=) | 7.338 | 0.53% | VUS | Johns Hopkins NGS |
| TINF2 | chr14:24240764T>G | c.716A>C | p.(His239Pro) | 0.054 | Not present | VUS | BMF XomeDxSlice GeneDx |
| WRAP53 | chr17:7703006C>T | c.1282C>T | p.(Leu428=) | 7.615 | Not present | VUS | BMF XomeDxSlice GeneDx |

*These variants were identified in the same patient. **The same variant was identified in two different individuals from this cohort. All variants were heterozygous.
