# Supplementary material for: Clinical and molecular correlates from a predominantly adult cohort of patients with short telomere lengths
Source: Blood Cancer J. 2021 Oct 22;11(10):170. doi: 10.1038/s41408-021-00564-7 (PMC8536738; doi:10.1038/s41408-021-00564-7)
Supplement: Supplementary file 4 — Supplementary table 3 [file 41408_2021_564_MOESM4_ESM.docx]

Supplementary table 3: Table showing dynamics of telomere length measurement in a single 47-year-old patient treated with bilateral lung transplant (in year 2016) for idiopathic interstitial pneumonia (fibrotic NSIP pattern) and emphysema at three different time-points 4 years post-transplant.

| **Date of FlowFISH** | **Lymphocytes** | | | **Granulocytes** | | |
| --- | --- | --- | --- | --- | --- | --- |
|  | **Absolute TL (kb)** | **Delta TL (kb)** | **Centile category** | **Absolute TL (kb)** | **Delta TL (kb)** | **Centile category** |
| 4/22/2020 | 5.23 | -1.23 | 1-10^th^ | 5.26 | -1.60 | 1-10^th^ |
| 10/13/2020 | 5.31 | -1.10 | >10^th^ | 5.92 | -0.91 | 10-50^th^ |
| 1/5/2021 | 5.05 | -1.36 | 1-10^th^ | 5.58 | -1.25 | 1-10^th^ |

Abbreviations: NSIP=Non-specific interstitial pneumonitis; FlowFISH= flow cytometry and fluorescence in-situ hybridization; TL=Telomere length.
