# Supplementary material for: Clinical and molecular correlates from a predominantly adult cohort of patients with short telomere lengths
Source: Blood Cancer J. 2021 Oct 22;11(10):170. doi: 10.1038/s41408-021-00564-7 (PMC8536738; doi:10.1038/s41408-021-00564-7)
Supplement: Supplementary file 5 — Supplementary table 4 [file 41408_2021_564_MOESM5_ESM.docx]

***Supplementary Table 4*:*** Table showing distribution of clinical features, genetic testing and outcomes for the considered FLOW FISH categories in the diagnostic assessment of STS (range or % only provided if n>1).

| **Characteristic (Median; % or range)** | **Total (n=233)** | **FLOW FISH TL centile categories (categorization possible in 233 patients)** | | | | | | ***P* value** |
| --- | --- | --- | --- | --- | --- | --- | --- | --- |
|  |  | **<1^st^ centile in both lymphocytes & granulocytes**  **(n=14)** | **<1^st^ centile in lymphocytes**  **> 1^st^ centile in granulocytes**  **(n=6)** | **<1^st^ centile in granulocytes**  **> 1^st^ centile in lymphocytes**  **(n=27)** | **1-10^th^ centile in both lymphocytes and granulocytes**  **(n=55)** | **1-10^th^ centile in lymphocytes, > 10^th^ centile in granulocytes**  **(n=56)** | **>10^th^ centile in lymphocytes &**  **> 1^st^ centile in granulocytes**  **(n=75)** |  |
| Age (in years) | 58 (4-83) | 50 (6-66) | 45 (5-61) | 48 (14-75) | 62 (4-77) | 60 (17-79) | 48 (10-83) | **0.0005** |
| No. of males (%) | 144 (57) | 9 (64) | 3 (50) | 16 (59) | 40 (73) | 30 (54) | 38 (51) | 0.2 |
| Family history | 63 (25) | 4 (29) | 1 (17) | 7 (26) | 22 (40) | 15 (27) | 14 (19) | 0.2 |
| Premature greying of hair (onset at age < 30 years) | 23 (10) | 3 (21) | 1 (17) | 4 (15) | 7 (13) | 4 (7) | 4 (5) | 0.3 |
| IIP | 135 (54) | 9 (64) | 3 (50) | 10 (37) | 48 (87) | 31 (55) | 31 (41) | **<0.0001** |
| Cytopenias | 103 (44) | 7 (50) | 1 (17) | 21 (78) | 19 (35) | 20 (36) | 35 (47) | **0.002** |
| Cirrhosis | 28 (12) | 1 | 1 | 4 (15) | 12 (22) | 3 (5) | 7 (9) | 0.1 |
| NRH | 4 (2) | 1 | 1 | - | - | - | 2 (3) | 0.09 |
| Immunodeficiency | 37 (16) | 2 (14) | 3 (50) | - | 4 (7) | 13 (23) | 15 (20) | **0.001** |
| *****Clinical likelihood of STS*** | | | | | | | | |
| Low (1) | 123 (53) | 7 (50) | 3 (50) | 12 (44) | 19 (35) | 31 (55) | 51 (68) | **0.009** |
| Intermediate (2) | 81 (35) | 4 (29) | 1 (17) | 12 (44) | 22 (40) | 22 (39) | 20 (27) | 0.3 |
| High (>2) | 29 (12) | 3 (21) | 2 (33) | 3 (11) | 14 (25) | 3 (5) | 4 (5) | **0.002** |
| Delta TL in lymphocytes (kb) | -1.12 (-5.11 to 2.8) | -2.73 (-5.11 to 2.62) | -2.62 (-3.28 to -2.02) | -2 (-2.49 to -0.6) | -1.45 (-2.47 to -0.7) | -0.975 (-2.43 to 1) | 0 (-0.99 to 2.8) | **<0.0001** |
| Delta TL in granulocytes (kb) | -1.27 (-10.6 to 6.35) | -2.76 (-4.1 to -1.9) | -1.4 (-2.34 to -0.81) | -2.69 (-4.7 to 2.29) | -1.6 (-10.6 to -0.86) | -1.14 (-2.5 to 5.72) | -0.3 (-2.7 to 6.35) | **<0.0001** |
| ***No. of patients with genetic testing | 73 (31) | 9 (64) | 2 (33) | 14 (52) | 16 (29) | 13 (23) | 19 (25) | **0.01** |
| *No. of patients with telomere-related variants* | | | | | | | | |
| Pathogenic/likely pathogenic | 9 (12) | 4 (44) | 1 | 2 (14) | 1 | 1 | - | **0.02** |
| VUS | 16 (22) | 2 (22) | - | 2 (14) | 3 (18) | 5 (38) | 4 (21) | 0.9 |

**Abbreviations:** FLOW FISH=flow cytometry fluorescence in-situ hybridization; IIP=idiopathic interstitial pneumonia; NRH=nodular regenerative hyperplasia; STS=short telomere syndrome; VUS=variant of uncertain significance. Significant clinical features for STS considered were personal history of premature greying of hair (onset at age < 30 years), IPF, unexplained cytopenias, cirrhosis or NRH, and unexplained immunodeficiency, or significant family history of the above (in one or more 1^st^ or 2^nd^ degree relatives). *This table includes data on 233 patients out of the total cohort of 252 patients. In 19 patients, data on both lymphocytes and granulocytes was not available for categorization into the TL categories. **Based on the number of the significant clinical features, clinical likelihood score was defined as low (1), intermediate (2), and high (>2). ***Included patients were tested with panels designed to test bone marrow failure-related genes. In other words, patients who underwent genetic testing with hematologic malignancy-based next generation sequencing panels were excluded.
