# Supplementary material for: Clinical and molecular correlates from a predominantly adult cohort of patients with short telomere lengths
Source: Blood Cancer J. 2021 Oct 22;11(10):170. doi: 10.1038/s41408-021-00564-7 (PMC8536738; doi:10.1038/s41408-021-00564-7)
Supplement: Supplementary file 6 — Supplementary figure 1 [file 41408_2021_564_MOESM6_ESM.docx]

**Figure 1S(A):** Figure showing overall survival (OS) analysis of patients with pulmonary disease who were treated with or without lung transplantation. Kaplan-Meier estimate of median OS did not statistically differ in patients treated with versus without lung transplant (30 months versus median not reached, *P*=0.4).

No transplant (n=82)

Lung transplant (n=19)

Log-rank test, *P*=0.3

**Figure 1S(B):** Figure showing overall survival (OS) analysis of patients with cytopenias who were treated with versus without allogeneic hematopoietic stem cell transplantation (HCT). Kaplan-Meier estimate of median OS did not statistically differ in patients treated with versus without HCT (median not reached in both groups, *P*=0.6).

Log-rank test, *P*=0.6

No HCT n=62)

HCT (n=6)
